# The influence of temperament and character profiles on specialty choice and well-being in medical residents

**DOI:** 10.7717/peerj.2319

**Published:** 2016-09-06

**Authors:** Martin Sievert, Igor Zwir, Kevin M. Cloninger, Nigel Lester, Sandor Rozsa, C. Robert Cloninger

**Affiliations:** 1 Department of Psychiatry, Washington University in St. Louis, Saint Louis, MO, United States; 2 Anthropedia Foundation, Saint Louis, MO, United States; 3 Departments of Psychiatry, Psychology, Genetics, Washington University in St Louis, Saint Louis, MO, United States

**Keywords:** Career choice, Medical specialization, Personality, Temperament, Character, Well-being

## Abstract

**Background:**

Multiple factors influence the decision to enter a career in medicine and choose a specialty. Previous studies have looked at personality differences in medicine but often were unable to describe the heterogeneity that exists within each specialty. Our study used a person-centered approach to characterize the complex relations between the personality profiles of resident physicians and their choice of specialty.

**Methods:**

169 resident physicians at a large Midwestern US training hospital completed the Temperament and Character Inventory (TCI) and the Satisfaction with Life Scale (SWLS). Clusters of personality profiles were identified without regard to medical specialty, and then the personality clusters were tested for association with their choice of specialty by co-clustering analysis. Life satisfaction was tested for association with personality traits and medical specialty by linear regression and analysis of variance.

**Results:**

We identified five clusters of people with distinct personality profiles, and found that these were associated with particular medical specialties Physicians with an “investigative” personality profile often chose pathology or internal medicine, those with a “commanding” personality often chose general surgery, “rescuers” often chose emergency medicine, the “dependable” often chose pediatrics, and the “compassionate” often chose psychiatry. Life satisfaction scores were not enhanced by personality-specialty congruence, but were related strongly to self-directedness regardless of specialty.

**Conclusions:**

The personality profiles of physicians were strongly associated with their medical specialty choices. Nevertheless, the relationships were complex: physicians with each personality profile went into a variety of medical specialties, and physicians in each medical specialty had variable personality profiles. The plasticity and resilience of physicians were more important for their life satisfaction than was matching personality to the prototype of a particular specialty.

## Introduction

Through popular media and opinions from those working in the medical profession itself, there are certain stereotypes about physicians that permeate medicine and mainstream culture (Thomas, 1997). With more physicians entering the profession from liberal arts and underserved backgrounds, the existence of only one archetype per specialty is extremely unlikely. Specialties vary greatly based on setting, amount of patient interaction, procedures involved, and daily variety.

Medical students often make a lifelong career decision based on limited exposure to each of these very different fields. These decisions are made based on a complex interaction of factors such as enjoyment of clinical rotations, standardized test scores, grades, earning potential, prestige, and general estimation of their cohesiveness with future colleagues (Cleland et al., 2012; Reed, Jernstedt & Reber, 2001). There is little quantifiable evidence used to help students make this major life decision. Most previous studies looked at personality differences between one specialty and all others or between large demographic groups like gender, practice setting, or provider levels (Drosdeck et al., 2015; Eley, Young & Przybeck, 2009; Hoffman, Coons & Kuo, 2010). A study that looked at a cohort of medical students was only able to compare between more common specialties based on the specialty choices of that class, so little is known about many of the smaller fields (Vaidya et al., 2004).

Variability in the vocational interests of people are known to be related to differences between the personalities of individuals in the general population (Hogan & Blake, 1999; Staggs, Larson & Borgen, 2007). The Temperament and Character Inventory (TCI) is particularly suitable for distinguishing physician’s vocational interests because it measures adaptive functioning as well or better than other modern inventories (Grucza & Goldberg, 2007) and distinguishes medical students and physicians who are interested in different medical specialties (El Sheikh et al., 2014; Eley, Young & Przybeck, 2009; Vaidya et al., 2004). The TCI measures seven dimensions of adaptive functioning, each of which are relevant for functioning and adaptability as a physician in such critical roles as decision making, human relationships, and emotional self-regulation (Eley et al., 2013). As described in Table 1, these traits include 3 aspects of character (mental self-government including Self-directedness, Cooperativeness, and Self-transcendence) and 4 aspects of temperament (emotional drives including Harm Avoidance, Novelty Seeking, Reward Dependence, and Persistence).

**Table 1:** Description of high and low scorers on temperament and character subscales of the Temperament and Character Inventory (TCI).

TCI scales	TCI subscales	High scorers	Low scorers
Novelty seeking			
	NS1 excitability	exploratory	reserved
	NS2 impulsivity	impulsive	rigid
	NS2 extravagance	extravagant	thrifty
	NS4 disorderly	rule-breaking	orderly
Harm avoidance			
	HA1 pessimism	pessimistic	optimistic
	HA2 fearfulness	fearful	risk-taking
	HA3 shyness	shy	outgoing
	HA4 fatigability	fatigable	vigorous
Reward dependence			
	RD1 sentimentality	sentimental	objective
	RD2 sociability	open	secretive
	RD3 attachment	friendly	detached
	RD4 dependence	approval-seeking	independent
Persistence			
	PS1 eagerness	enthusiastic	hesitant
	PS2 hard-working	determined	easily discouraged
	PS3 ambition	ambitious	lazy
	PS4 perfectionism	perfectionistic	underachieving
Self-directedness			
	SD1 responsibility	responsible	blaming
	SD2 purposefulness	purposeful	aimless
	SD3 resourcefulness	resourceful	helpless
	SD4 self-acceptance	hopeful	hopeless
	SD5 self-actualizing	self-actualizing	unfulfilled
Cooperativeness			
	CO1 social tolerance	tolerant	prejudiced
	CO2 empathy	empathetic	self-centered
	CO3 helpfulness	considerate	hostile
	CO4 compassion	forgiving	revengeful
	CO5 conscience	principled	opportunistic
Self-Transcendence			
	ST1 self-forgetfulness	acquiescent	controlling
	ST2 transpersonal identification	altruistic	individualistic
	ST3 spiritual acceptance	spiritual	skeptical

We hypothesized that there would be significant differences in TCI profiles between resident physicians in various specialties at a large training hospital in the Midwestern United States. We expected procedure-based specialties (emergency medicine and surgical specialties) to be higher in novelty seeking compared to other fields (Vaidya et al., 2004). We expected to find lower sociability and self-transcendence in specialties with lower patient interaction (pathology and radiology) than primary care and psychiatry. Given the recent data on physician burnout rates (Shanafelt et al., 2015), we also wanted to look at how personality might impact life satisfaction. We expected physicians whose personalities were outliers in their specialty to have lower life satisfaction due to a dissonance between their personality and the demands of the job, as has often been suggested in studies of person-environment congruence for vocational behavior in general (Edwards et al., 2006; Judge et al., 2005; Spokane, Meir & Catalano, 2000).

## Materials and Methods

### Participants

All physicians in residency or fellowship at Barnes-Jewish Hospital and St. Louis Children’s Hospital during the 2015–2016 academic year were asked to participate in the study through email in July 2015. These hospitals are the main training sites for Washington University in St. Louis. The recruitment email was sent through the Graduate Medical Education office in accordance with internal protocols for research requests of house staff. Two additional emails requesting participation were sent in two-month increments. All residency directors also received an additional email from the research team requesting time to speak about the study to potential responders during a residency meeting of their choice. Only general surgery, urology and psychiatry residents received a description of the study in person.

Fifty-nine percent of respondents were female. Average age was 29.7 years old (range 24–39). Using ACGME data, we estimated that there were 916 residents who were eligible for the study. Using this total, we obtained an 18.45% completion rate for all specialties (169/916). This varied by specialty from 4.76% for neurosurgery to 40% for pediatric infectious disease. One responder did not report their specialty but the remaining 168 surveys were complete and used for clustering analysis. There were an additional 65 surveys that were incomplete and were excluded.

### Human subjects approval

The Internal Review Board at Washington University Medical School in St. Louis gave approval for the study (#201503022) on 3/23/2015.

### Assessment instruments

The emails that participants received included a link to the study materials that they could access from any computer (Surveymonkey©). Clicking the link showed an informed consent document with explanation of the study. Participants were then asked to enter demographic information (initials, age, gender, specialty, ethnicity, and training level). The Temperament and Character Inventory-revised (TCI-R) and the Satisfaction with Life Scale (SWLS) were then given in succession.

The TCI-R is a self-report questionnaire answered on a 5-point Likert scale (1 = definitely false to 5 = definitely true) that assesses four temperament dimensions and three character dimensions (Cloninger, Svrakic & Przybeck, 1993; Goncalves & Cloninger, 2011). The temperament dimensions measure a person’s basic emotional drives, including Novelty Seeking (NS), Harm Avoidance (HA), Reward Dependence (RD), and Persistence (P), as described in Table 1. The character dimensions measure a person’s goals and values, including Self-directedness (SD), Cooperativeness (CO), and Self-Transcendence (ST), as described in Table 1. The short form with 140 items is a valid and reliable measure of its subscales for group research like this project (Zohar & Cloninger, 2011), and was used to reduce subject burden. To provide a comparison to people in the general population, the Z-scores of physicians for each of the dimensions of the TCI-140 were computed on the basis of the mean and standard deviation of scores in the normative general population, as described elsewhere (Cloninger et al., 1994; Zohar & Cloninger, 2011). Descriptors for physicians were based on ranking in each successive one-sixth of the general population: lowest one-sixth (very low, 1st to 16th percentile), second (low, 17–33 percentile), third (low average, 34–50 percentile), fourth (high average, 51–67 percentile), fifth (high, 68–83 percentile), and highest one-sixth (very high, 84–99 percentile), as described elsewhere (Cloninger et al., 1994).

The SWLS is a five question self-report questionnaire answered with a Likert scale (1 = strongly disagree to 7 = strongly agree). The sum of all 5 questions is combined for a total score that is a valid and reliable measure of subjective life satisfaction (Diener et al., 1985; Pavot et al., 1991). The qualitative level of satisfaction for each person varies from very high (total score 31–35, average 6.2–7.0), high (total 26–30, average 5.2–6), slightly high (total 21–25, average 4.2–5), neutral (total 20, average 4), slightly low (total 15–19, average 3–3.8), low (total 10–14, average 2–2.8), to very low (total 5–9, average 1–1.8). Individual results were not provided to each participant because that would have required us to identify them individually.

### Statistical analysis

#### Cluster analysis

**Figure 1:**
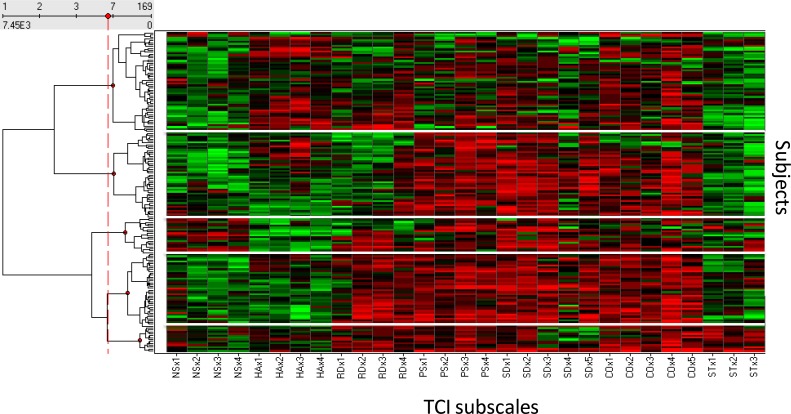
Identifying clusters of subjects sharing similar TCI subscale scores. Clusters 1–5 appear in sequence from top (cluster 1) to bottom (cluster 5). Ward’s clustering method was used and calculated the incremental sum of squares. The similarity measure was the Half square Euclidean distance. The optimal number of 5 clusters was calculated using the Davies–Bouldin validity index.

The average value per item for each subscale was computed in preparation for cluster analysis (Cloninger et al., 1994). The medical specialties of respondents were coded hierarchically according to their training path, such as pediatrics followed by pediatric subspecialties. Then we carried out hierarchical agglomerative clustering (Statistical Toolbox, Matlab 2007b, Spotfire Decision Site 9.1.2, Fig. 1) using Ward’s linkage method and Half Square Euclidean distance as the similarity measure to group subjects sharing similar TCI subscale scores (Bezdek, 1998). This is a crisp clustering method in which all subjects are assigned to a cluster and each subject is assigned to only one cluster. The number of clusters was determined by the Davies and Bouldin validity index (Bezdek, 1998; Zwir, Huang & Groisman, 2005) to identify the optimal number of clusters. Prototypes or centroids of a cluster are calculated as the average value of each subscale within a cluster. The function that controls the vertical order in which a row is plotted in the hierarchical clustering procedure (Spotfire Decision Site 9.1.2) (Arnedo et al., 2013).

We checked for significant overlap among clusters of subjects with similar TCI subscales and clusters of subjects with similar specialties using Hypergeometric statistics (Arnedo et al., 2013; Zwir, Huang & Groisman, 2005). This allowed us to characterize co-clusters (i.e., relationships among groups of people). The degree of overlap between two clusters was assessed by calculating the pair-wise probability of intersection among them based on the Hypergeometric distribution (*PI*_hyp_):

\begin{eqnarray*}P{I}_{hyp}({P}_{i},{G}_{j})=1- \sum _{q=0}^{p-1} \left( \begin{array}{@{}c@{}} \displaystyle h\\ \displaystyle q \end{array} \right) \left( \begin{array}{@{}c@{}} \displaystyle g-h\\ \displaystyle n-q \end{array} \right) \left/ \right. \left( \begin{array}{@{}c@{}} \displaystyle g\\ \displaystyle h \end{array} \right) \begin{array}{@{}c@{}} \displaystyle h={|}{P}_{j}{|}\\ \displaystyle n={|}{G}_{j}{|}\\ \displaystyle p={P}_{j}\cup {G}_{j} \end{array}\quad \quad \text{(Arnedo et al., 2013)} \end{eqnarray*}

where *p* observations belong to a set *P*_*i*_ of size *h*, and also belong to a set *G*_*j*_ of size *n*; and *g* is the total number of observations. Therefore, the lower the *PI*_hyp_, the higher the extent of overlap. The *p*-value of the hypergeometric test is used here as a measure of association strength.

#### Relations of life satisfaction to personality and medical specialty

Variability in SWLS scores was examined in relation to individual TCI traits by Pearson correlations and multiple linear regression using standard methods. The source of the variability in SWLS scores among TCI clusters and medical specialties associated with them was also examined using the standard ANOVA (Statistical Toolbox, Matlab 2007b). We considered a matrix composed of the TCI clusters as columns (i.e., sources of explanatory variability), where the rows were composed of the average SWLS scores of members of each TCI cluster (i.e., error variance). After ANOVA, we performed multiple pair-wise *t*-test comparisons with a significance level cutoff value of 0.05 (Multcompare, Statistical Toolbox, Matlab 2007b).

## Results

### Comparison to the general population

The distribution of TCI scores in the whole sample was analyzed to compare the physicians to the general population. The means and standard deviations of physician’s Z-scores are shown in Table 2. On average the physicians were only extreme in being very high in TCI Persistence. On average, they were slightly high in each of the three character dimensions, low in Reward Dependence, and slightly low in Novelty Seeking and Harm Avoidance. However, there was substantial variability around these mean values, which was examined by cluster analysis.

**Table 2:** Scores on the TCI for the whole physician sample as *Z*-scores based on normative population parameters (*n* = 169).

TCI dimension	Q ualitative descriptor	Percentile mean	*Z*-score mean	*Z*-score standard deviation
Novelty seeking	Low average (slightly orderly)	38.6	−0.29	1.20
Harm avoidance	Low average (slightly optimistic)	46.0	−0.10	1.20
Reward dependence	Low (detached)	21.2	−0.80	1.00
Persistence	Very high (very determined)	88.5	+1.20	1.00
Self-directedness	High average (slightly responsible)	65.5	+0.40	0.97
Cooperativeness	High average (slightly helpful)	59.9	+0.25	1.20
Self-transcendence	High average (slightly altruistic)	55.2	+0.13	0.99

### Cluster analysis

Clusters of subjects sharing similar TCI subscale profiles were identified using Ward’s clustering method without regard for the medical specialty of the physicians (Fig. 1, Table S1). TCI dimensions were reported from low to high based on the average Likert scale score for each trait, as depicted by color-coding in Fig. 1 for ease of visual inspection and pattern recognition. The optimal number of personality clusters identified was five according to the Davies–Boudin validity index (Fig. 1). Each of these five clusters was characterized by a distinct personality profile.

**Figure 2:**
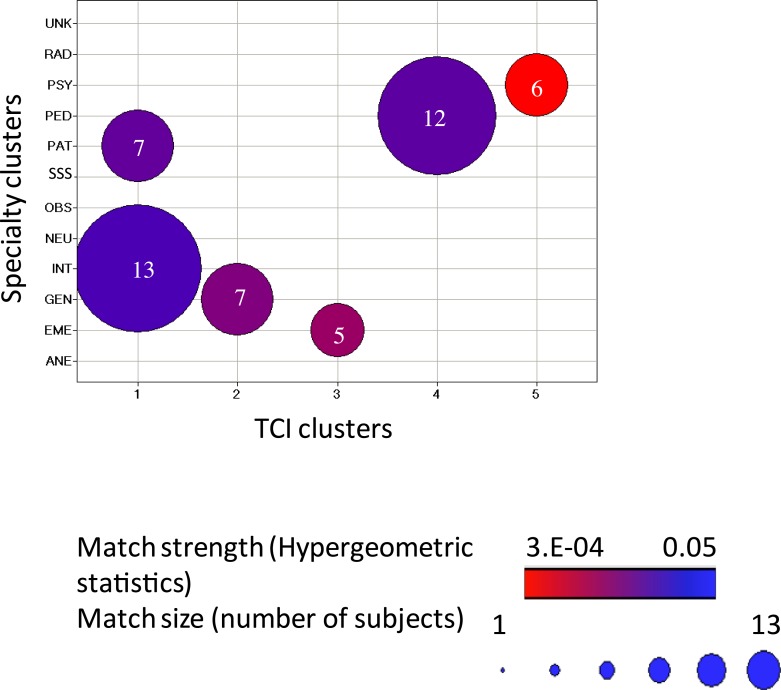
The match between TCI clusters and physician’s specialties. The cluster interaction was calculated using Hypergeometric statistics. *P*-values < 0.05 were reported and color coded (red: low, blue: high), so the red circle indicated the strongest association (i.e., lowest *p* value) was between cluster 5 and psychiatrists. The size of the circles indicates the number of subjects in the intersection.

**Figure 3:**
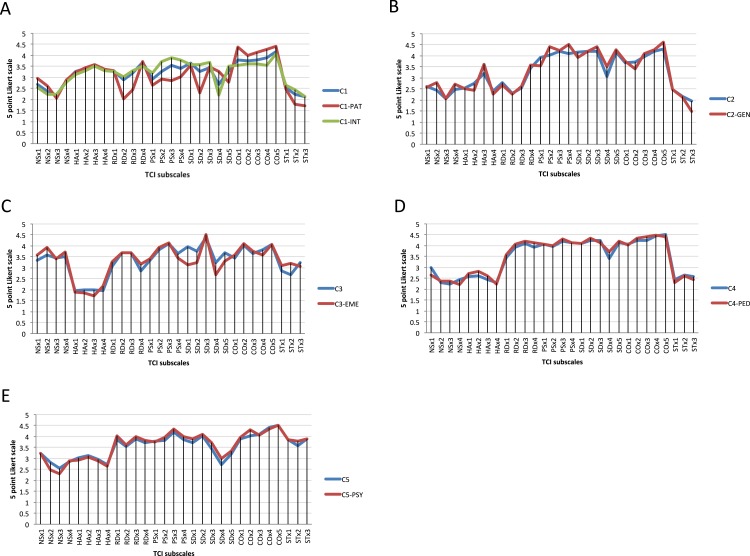
Prototypes (centroid, i.e., profile of average values) of the TCI clusters in comparison to those of associated specialties. The *X*-axis corresponds to the TCI subscales. *Y*-axis corresponds to the TCI in its 5 point Likert scale on which 1 indicates definitely false and 5 indicates definitely true. (A) Prototype of cluster 1. The centroid calculated using all subjects in the cluster is color-coded in blue. The centroid calculated from the intersection of subjects in cluster 1 and the specialty of pathology is color-coded in red. The centroid calculated from the intersection of subjects in cluster 1 and internal medicine is color-coded in green. (B) Prototype of cluster 2. The centroid calculated using all subjects in the cluster is color-coded in blue. The centroid calculated from the intersection of subjects in cluster 2 and General Surgery is color-coded in red. (C) Prototype of cluster 3. The centroid calculated using all subjects in the cluster is color-coded in blue. The centroid calculated from the intersection of subjects in cluster 3 and Emergency Medicine is color-coded in red. (D) Prototype of cluster 4. The centroid calculated using all subjects in the cluster is color-coded in blue. The centroid calculated from the intersection of subjects in cluster 4 and Pediatrics is color-coded in red. (E) Prototype of cluster 5. The centroid calculated using all subjects in the cluster is color-coded in blue. The centroid calculated from the intersection of subjects in cluster 5 and Psychiatry is color-coded in red.

Next the five personality clusters were tested for match with the physician’s medical specialty (Fig. 2, Table S2). Each cluster was more common in a particular specialty except for cluster 1 which was associated with two specialties. Each specialty had various personality types within it and each cluster was not exclusively found in one specialty, as discussed and illustrated in detail in ‘Novel Findings’. The prototypes of each personality-based cluster are depicted in Fig. 3, along with the prototype of each associated medical specialty for comparison (Figs. 3A–3E)

Cluster 1 (“investigative”) was most common in pathology and internal medicine (Figs. 2 and 3A). Of responding pathologists and internists, 58% (7/12) and 43% (13/30) fit this cluster, respectively. This cluster had a temperament profile with prominent orderliness (i.e., low NS) accompanied by average HA, average sociability (i.e., average RD except for the subscale for dependence which was higher), and average persistence. The character profile of this cluster was purposeful (i.e., average SD), highly collegial (i.e., high CO) and highly skeptical (i.e., low ST). Within this cluster, the pathologists who were closely associated with this profile and were lower in warm communication (RD2), goal setting (SD2) and persistence (P) compared to the internists but had higher average CO. Overall, people with this TCI profile can be described as orderly, organized, and skeptical investigators who like to test and verify facts.

Cluster 2 (“commanding”) was most common in general surgery, occurring in 58% (7/12) of respondents from that specialty (Figs. 2 and 3B). This cluster had a temperament profile with prominent indicators of “independence” and autonomy, including low NS, low components of HA with higher scores in shyness (HA3), low components of RD except for dependence (RD4), and very high *P*. People with this cluster were highly self-directed, and, like strong leaders, promote cooperativeness (i.e., high CO) as long as they remain in charge. They were also very low in ST, so they were skeptical and controlling like a commander, rather than a collegial peer.

Cluster 3 (“rescuing”) was most common in emergency medicine, occurring in 38% (5/13) of the respondents from that specialty (Figs. 2 and 3C). The most unique cluster, it had a passionate temperament: they thrive on novelty and excitement (i.e., high NS) without fear of facing dangerous situations (i.e., very low HA) and had average sociability (average RD overall but warm in communication and high in attachment). They were also persevering (i.e., high *P*) and particularly ambitious. Their character profile was organized with high SD (especially high in resourcefulness), high CO and average ST. People in a subgroup of this cluster were impulsive with low responsibility (low SD1) and higher novelty seeking (NS).

Cluster 4 (“dependable”) was most common in pediatricians, occurring in 35% (12/34) of the respondents from that specialty (Figs. 2 and 3D). They had a trustworthy temperament (i.e., reliable with low NS, low HA, high RD (with especially high warm communication), and were highly persevering (i.e., very high *P*). For character traits, they were rather controlling (i.e., very high SD, very high CO and low ST), but were more cooperative and encouraging than those in the “commanding” cluster 2 typical of surgeons. In other words, they are encouraging and dependable guides to others.

Cluster 5 (“compassionate”) was most common in psychiatrists, occurring in 38% (6/16) of the respondents from that specialty (Figs. 2 and 3E). This cluster was the most strongly associated with a particular specialty (Fig. 2, Table S2). Physicians in this cluster had a sociable temperament: they had average NS, average HA, high RD (very high in sentimentality), and high *P*. Uniquely, this cluster had high values in all three character traits- high SD, very high CO and high ST, which is described as a creative character profile. Because they are self-transcendent and highly compassionate (CO4), they are most distinguished by their compassion.

### Life satisfaction and its relationship with personality

When analyzing SWLS scores and their relationship to TCI dimensions, we found a correlation structure typical of the general population (Table 3). SWLS scores were correlated moderately (*p* < 0.01) with Self-directedness (*r* = 0.62), Persistence (*r* = 0.38), and Harm Avoidance (*r* = − 0.27), life satisfaction was also weakly but significantly correlated with Cooperativeness (*r* = 0.18) and Reward Dependence (*r* = 0.16), but not with Novelty Seeking or Self-transcendence.

**Table 3:** Correlations among TCI dimensions and Satisfaction with Life Scale Scores.

	NS	HA	RD	PS	SD	CO	ST
Novelty seeking							
Harm avoidance	−0.53						
Reward dependence	0.09	−0.06					
Persistence	−0.19^*^	−0.30^**^	0.00				
Self-directedness	−0.15	−0.42^**^	0.10	0.51^**^			
Cooperativeness	−0.14	0.15	0.45^**^	0.08	0.33^**^		
Self-transcendence	0.22^**^	0.11	0.16^*^	0.13	−0.10	0.06	
Satisfaction with Life	−0.11	0.27^**^	0.16^*^	0.38^**^	0.62^**^	0.18	0.01

**Notes.**

* Correlation is significant at the 0.05 level (2-tailed).

** Correlation is significant at the 0.01 level (2-tailed).

Multiple linear regression was carried out to predict SWLS scores from all 7 TCI dimensions, age, gender, and years of postgraduate education. Self-directedness was the only significant predictor of life satisfaction (*R*^2^ = 0.413) in the multiple regression analysis. There were no significant differences in satisfaction among ethnic groups.

**Figure 4:**
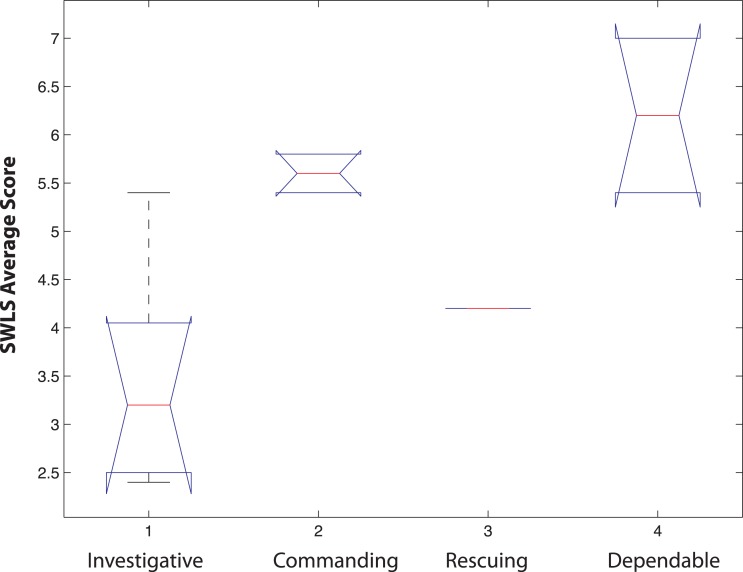
Box plots of the distribution of average Satisfaction with Life Scale (SWLS) scores in physicians comprising each of the 5 TCI clusters. In each box, the central mark is the median, the edges of the box are the 25th and 75th percentiles, the whiskers extend to the most extreme data points not considered outliers, and outliers are plotted individually (Statistical Toolbox, Matlab 2007b). According to ANOVA (Statistical Toolbox, Matlab 2007b), Clusters 2, 4 and 5 have means significantly different than Cluster 1 (*p* < 0.05). Clusters 2 and 4 have means significantly different than Cluster 3 (*p* < 0.05). Finally Clusters 2, 4 and 5 have similar means.

We also examined the variability in life satisfaction scores among the 5 TCI clusters of physicians. The means and distributions of life satisfaction scores are shown in Fig. 4. There was significant variability in the means of the 5 clusters overall according to ANOVA (sum of squares 47.02 with 4 degrees of freedom, mean squares 11.75, *F* = 10.47, *p* = 1.41 E−07). Clusters 2, 4 and 5 had means significantly different than Cluster 1 (pair-wise *t*-tests, *p* < 0.05). Clusters 2 and 4 have means significantly different than Cluster 3 (pair-wise *t*-tests, *p* < .05). Finally Clusters 2, 4 and 5 have similar means, as shown in Fig. 4.

The life satisfaction scores of physicians in different specialties were also examined. The differences between specialties in life satisfaction were only significant between psychiatry vs pathology and surgical subspecialties vs pathology (Table 4). Satisfaction scores between specialties likely differed based on traits that are known to play a large role in satisfaction in the general population, such as SD. Most specialty satisfaction means were in the (moderately) high satisfaction range (i.e., total score = 26–30, average score 5.2–6.0). Pathology, emergency medicine, and radiology were in the slightly high satisfaction range (total score = 21–25, average score 4.2–5.0).

**Table 4:** Means and standard deviations of SWLS scores by specialty.

Specialty	*N*	Qualitative mean level of life satisfaction^*^	Mean score	Std. dev	Std. error	Minimum	Maximum
Unknown (UNK)	1	*Very high*	31	–	–	31	31
Surgical Subspecialties (SSS)^*^	15	*High*	29.93	6.006	1.551	11	35
Obstetrics-gynecology (OBS)	7	*High*	29.43	6.399	2.419	16	35
Psychiatry (PSY)^*^	16	*High*	28.81	4.902	1.225	14	35
Internal medicine (INT)	30	*High*	27.68	4.743	0.852	17	34
Neurology (NEU)	8	*High*	27.38	4.838	1.711	19	34
Anesthesiology (ANE)	9	*High*	27.33	4.387	1.462	22	35
Pediatrics (PED)	34	*High*	27.00	4.789	0.834	10	35
General surgery (GEN)	12	*High*	26.17	5.441	1.571	17	32
Radiiology (RAD)	12	*Slightly high*	25.67	7.524	2.172	8	32
Emergency Medicine (EME)	13	*Slightly high*	24.62	7.5	2.08	10	34
Pathology (PAT)^*^	12	*Slightly high*	21.58	7.44	2.148	12	35
Total	169	*High*	26.99	5.866	0.451	8	35

**Notes.**

* Mean scores are interpreted according to mean values as very high (31–35), moderately high (26–30), slightly high (21–25), neutral (20), slightly low (15–19), low (10–14), and very low (5–9) based on Diener’s recommended descriptions. Our sample of physicians was more satisfied than people in the general population. Among the various specialties, physicians in surgical subspecialties and in psychiatry were significantly more satisfied than pathologists (*p* < 0.05).

**Figure 5:**
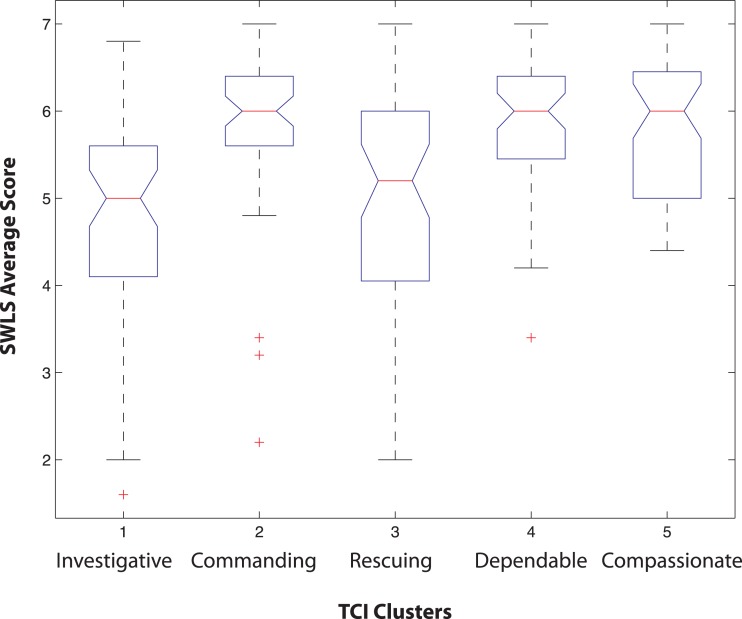
Boxplots of the distribution of average Satisfaction with Life Scale scores for physicians in pathology who have various TCI profiles. Pathologists in cluster 1 (“investigative”) were significantly (*p* < 0.05) lower than those in cluster 4 (“dependable”).

In order to assess the role of person-environment congruence on life satisfaction, we examined the life satisfaction scores of physicians in each of the six medical specialties that were associated with a particular one of the five TCI clusters using ANOVA (Table 5). Physicians in pathology and in emergency medicine varied significantly (*p* < 0.05) in their SWLS scores depending on their TCI cluster (Table 5). Pair-wise *t*-tests showed that the direction of the differences were not in the direction predicted by personality-specialty congruence (i.e., matching the cluster associated with one’s chosen specialty): pathologists with TCI cluster 1 (“investigative”, which is associated with the choice of pathology as a specialty) were significantly *lower* in life satisfaction than those with TCI cluster 4 (“dependable”) (*p* < 0.05) (Table 4), as illustrated in Fig. 5. Likewise, emergency medicine physicians with TCI cluster 3 (“rescuing”, which is associated with the choice of emergency medicine as a specialty) were *lower* in life satisfaction than those with TCI cluster 2 (“commanding”) (*p* < 0.05).

**Table 5:** Variation in average Satisfaction with life scores (SWLS) per item (total score /5) according to the 5 TCI personality clusters among physicians in each of the 6 medical specialties that were significantly associated with a TCI cluster.

Specialty (total *n*)			Average SWLS per item			ANOVA *F* statistic (*p*-value)
	Cluster 1	Cluster 2	Cluster 3	Cluster 4	Cluster 5	
Pathology (*n* = 12)	3.43^*^ (*n* = 7)	5.60 (*n* = 2)	4.20 (*n* = 1)	6.20^*^ (*n* = 2)	– (*n* = 0)	**5.03** (*p* < 0.05)
Internal Medicine (*n* = 30)	5.14 (*n* = 13)	6.00 (*n* = 5)	5.20 (*n* = 2)	5.98 (*n* = 8)	4.90 (*n* = 2)	1.79 (NS)
General Surgery (*n* = 12)	5.00 (*n* = 1)	5.66 (*n* = 7)	4.40 (*n* = 2)	3.40 (*n* = 1)	6.00 (*n* = 1)	1.82 (NS)
Emergency Medicine (*n* = 13)	5.07 (*n* = 3)	5.88^*^ (*n* = 5)	3.88^*^ (*n* = 5)	– (*n* = 0)	– (*n* = 0)	**2.98** (*p* < 0.05)
Pediatrics (*n* = 34)	4.95 (*n* = 12)	5.68 (*n* = 8)	5.20 (*n* = 1)	5.67 (*n* = 12)	7.00 (*n* = 1)	1.88 (NS)
Psychiatry (*n* = 16)	5.30 (*n* = 2)	6.00 (*n* = 4)	4.90 (*n* = 2)	5.80 (*n* = 2)	6.03 (*n* = 6)	0.60 (NS)

**Notes.**

* *p* value <0.05 by paired *t*-test in bold.

## Discussion

### Novel findings

We found that physicians as a group are moderately resilient and healthy in their functioning, but there is still substantial diversity in their temperament and character profiles. We made two novel and important observations about the personality of physicians in this study: first, there are strong associations between a physician’s personality profile and their choice of medical specialty, and second, having a personality profile associated with a particular specialty may lead to lower, not higher, life satisfaction. Both of these findings point to the importance of diversity of personality among physicians in various specialties and to a greater importance of flexibly adapting to one’s chosen specialty rather than trying to choose a specialty in which the personality prototype is similar to one’s own personality.

**Figure 6:**
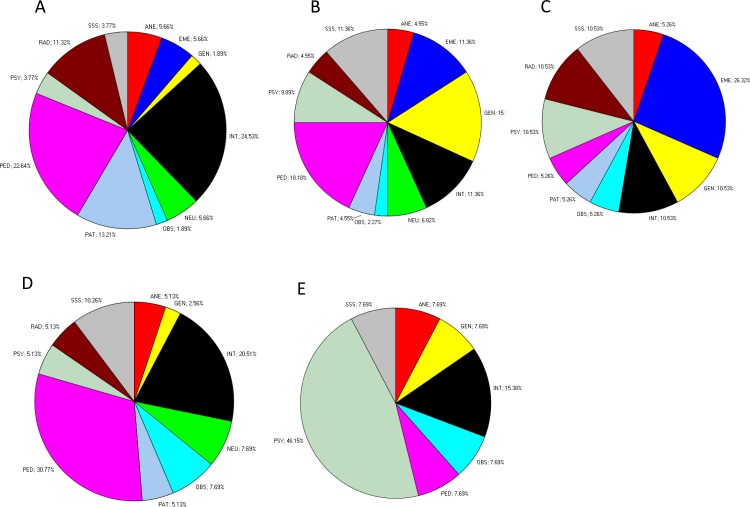
Pie chart showing the occurrences of specialties (%) in each TCI cluster. Specialties corresponding to TCI cluster (A) 1, (B) 2, (C) 3, (D) 4, and (E) 5.

Although we found strong associations between clusters of physicians with distinct personality profiles and their choice of medical specialty, the relationships between personality profiles and specialty choice were complex. In other words, the relations were “many-to-many”, not “one-to-one”. We observed that multiple personality profiles occurred among physicians within each specialty and that physicians in each of the personality clusters chose to enter a variety of specialties. Although the associations were strong, there is not just one personality profile that is consistently found in each particular specialty. The wide range of medical specialties observed in physicians in each of the five TCI personality clusters is shown in Fig. 6 to make this diversity clear and concrete.

The choice of a medical specialty does appear to involve, at least in part, a matching of one’s interests and strengths to the working conditions and opportunities of particular specialties (Reed, Jernstedt & Reber, 2001). Accordingly, the TCI could be used to assist physicians in identifying specialties that may match their interests because vocational interests and career choices are widely understood to be expressions of personality in general (Hogan & Blake, 1999; Staggs, Larson & Borgen, 2007) and in medicine in particular (El Sheikh et al., 2014; Eley, Young & Przybeck, 2009; Vaidya et al., 2004). However, our findings indicate that congruence between one’s personality and that most strongly associated with choice of a particular specialty does not consistently lead to greater life satisfaction. Adaptability appears to be more important for the well-being of physicians than person-environmental matching.

### Resilience and personality

All five clusters had relatively high scores on the traits characteristic of high levels of resilience, plasticity, and well-being (high SD, CO, PS, and low HA) (Cloninger & Cloninger, 2011; Cloninger & Zohar, 2011; Eley et al., 2013). Self- directedness is the ability to understand how personal actions influence outcomes so high scores in this trait indicate responsibility, maturity, and the ability to admit and learn from mistakes. Cooperativeness is the ability to function in a group with other people so high scores indicated tolerance, empathy and compassion. Persistence was also relatively high in all five clusters. Persistence is the ability and willingness to keep working hard for success despite frustration and fatigue. High scores in *P* are self-selected by the long educational training involved in medicine but can also signify perfectionism, which can predispose people to strained relationships or career burnout (Cloninger et al., 2012). Low scores in HA indicate optimism and ability to remain calm in risky situations. Burnout occurs in all specialties at alarming rates (Shanafelt et al., 2015) so identifying its causes and potential solutions are paramount.

### Diversity in personality profiles of medical specialists

The five clusters of physicians that we identified had very distinctive personality profiles. The physicians in “investigative” Cluster 1 were highly intellectual, inquisitive, and liked to test things in a systematic way, as indicated by their being highly orderly (i.e., low NS) and skeptical (i.e., low ST). This profile was most common in pathology and internal medicine with slight differences between those two subgroups. The people in the investigative cluster were somewhat socially reserved (i.e., average RD overall) but did want approval and cooperation in interactions with others (i.e., high RD4 and CO), as has been reported for pathologists in Egypt using the TCI-R (El Sheikh et al., 2014). The high cooperativeness scores may reflect the amount of interaction they have with other physicians and the need for collaboration on diagnoses with various specialties. This cluster fits with the work demands of pathology requiring less warm communication with patients and more of a focus on objective data, which is similar to past findings about specialties that require less patient interaction (Reed, Jernstedt & Reber, 2001). Internal medicine also has a grouping in this cluster which was less strong in its association. This subgroup of internists has relatively higher warm communication and persistence compared to their pathology colleagues. Several studies using different assessment tools found similar findings in internists, such as a tendency to be skeptical, prefer working with things over people, lower extraversion scores and high persistence (Borges & Savickas, 2002).

The physicians in the “commanding” Cluster 2 described themselves as skeptical, assertive and objective. This profile was commonly associated with the choice of general surgery as a specialty. They are independent and like being in control (i.e., high SD), which is useful when making quick decisions under stressful circumstances. They are highly skeptical (i.e., very low self-transcendence). Their high persistence and high self-directedness are likely self-selected by the long hours and physical demands of the job (Thomas, 1997). Their high cooperativeness is a good match to the demands for cooperative teamwork in surgery and the collaboration required with reduced residency work-hours. The “surgical personality” has been addressed more than most fields: one study found that most doctors believed surgeons shared certain traits that were often negative stereotypes (Thomas, 1997). A survey asking general surgery residents and attending physicians to rank descriptors of ideal surgeons found the most agreement for admits error, well disciplined, considers all facts, highly motivated, consistent, and listens, which are all traits that indicate self-sufficiency and internal discipline (Greenburg, McClure & Penn, 1982). A study of graduate medical students using the TCI-R found that those in surgery were more self-directed, particularly more resourceful (SD3), than others (El Sheikh et al., 2014), just as we observed. Some past studies have noted group differences such as high NS in surgeons (Vaidya et al., 2004), which we did not find in this cluster but may occur in some surgeons elsewhere. The personality traits of surgeons have also been found to vary based on experience level (Borges & Savickas, 2002; Drosdeck et al., 2015) and we only surveyed resident physicians.

The physicians in “rescuing” Cluster 3 described themselves as passionate and thriving on novelty and unpredictable situations. These physicians were likely to choose a career in emergency medicine. This is somewhat consistent with prior research finding high novelty seeking in procedure-based specialties (Vaidya et al., 2004). Because the ER involves many novel and unpredictable situations, a wide variety of procedures under difficult circumstances, the low HA and high NS of physicians in this cluster appear to be an excellent match for the specialty of emergency medicine and perhaps rural medicine (Eley et al., 2013). They are high in various RD subscales that reflect the interpersonal strengths of managing patients and families in extremely dire circumstances. High persistence and cooperativeness are helpful in a stressful environment that requires interactions with almost every specialty under tense conditions. Burnout rates are extremely high in emergency medicine- reaching 65% in residents (Shanafelt et al., 2015; Takayesu et al., 2014). Intolerance of uncertainty was correlated to burnout in emergency medicine (Takayesu et al., 2014) so more work remains to be done to see if people in our identified cluster are less likely to suffer burnout due to low HA scores.

The physicians in “dependable” Cluster 4 described themselves as reliable, warmly supportive, nurturing and conscientious. This personality profile was strongly associated with the choice of pediatrics as a specialty. Pediatricians serve a role in modeling parental behavior, which is a good match for the trust-inspiring features of people with this warm and comforting archetype. Being very high in persistence and self-directedness helps with their high workload and dealing with emotionally difficult situations. Very high cooperativeness is useful on collaborative rounds and interactions with multiple consulting subspecialists. Previous research into pediatrician personality trends is limited (Borges & Savickas, 2002).

The physicians in “compassionate” Cluster 5 described themselves as sociable, altruistic, and self-transcendent. This personality profile was strongly associated with the choice of psychiatry as a specialty. This profile is unique for having high scores in all three character traits, which indicates intuition and creativity. These high character scores are helpful when treating severe emotional problems. Often seen as treating the most destitute patients with medications and psychotherapies that are somewhat limited in efficacy, this cluster reflects physicians who are benevolent and sympathetic. This is consistent with literature categorizing psychiatry residents as sympathetic, trusting, cooperative and altruistic (Borges & Savickas, 2002; Chowdhury et al., 1987). These are useful traits in a field where the doctor-patient interaction is crucial for improving outcomes (Horvath, 2015). It is remarkable that the physicians choosing psychiatry in this sample were highly humanistic even though the psychiatry residency of the institution involved in this study is known to emphasize the medical model and neuroscience in its approach.

### Implications for specialty selection and health promotion in physicians

In an extremely competitive medical school application process, having evidence that certain personality profiles are more likely to succeed could be helpful for selection committees (Bore, Munro & Powis, 2009). We suspect only those low in all character traits and persistence would have universal difficulty in completing training. Preserving the remaining individual variation is likely to be important to keep medicine inclusive and heterogeneous, as well as providing the diversity needed to adapt to the wide variety of specialized skills that comprise modern medical practice. Personality assessment as a screening procedure could also help protect applicants from being graded poorly on an interview by faculty who may prefer to screen people with particular personality styles similar to their own (Brothers & Wetherholt, 2007).

For medical students, making a career choice with only two years of clinical experience is often difficult. More information about how an applicant’s personality structure would match with a certain specialty or that work environment could be valuable for that decision (Drosdeck et al., 2015; Hoffman, Coons & Kuo, 2010; Vaidya et al., 2004).

Prior work has suggested that the success of physicians in particular specialties (Foster et al., 2010) and their burnout rates (Shanafelt et al., 2015) may depend on their personality profile. However, success in residency can be difficult to measure (Ozuah, 2002; Smith, 1993). Many of our subjects were just beginning their specialty training, so we measured their overall life satisfaction, which allowed comparisons across all levels of specialty training. Congruence between one’s interests and personality (“person-environment congruence” or “person-environment fit”) are often assumed to lead to greater job and/or life satisfaction, but in fact the relationships observed have been weak and inconsistent (Edwards et al., 2006; Spokane, Meir & Catalano, 2000), as is typical of the complexity we observed. In fact, we found that physicians whose personality were congruent with those associated with pathology and with emergency medicine had lower life satisfaction than those with personality profiles that were atypical of those specialties (Table 4 and Fig. 5). These two specialties were also slightly but significantly lower in their life satisfaction than physicians choosing other specialties (Fig. 4). However, it is possible that the lower satisfaction of these individuals may be attributable to slight differences in personality that were not fully captured by the five average cluster profiles. The pathologists in the investigative cluster were slightly less sociable (lower RD2 and RD3) and less certain about their goals in life (lower SD2) than others in the same cluster (see Fig. 3A). Likewise the emergency medicine physicians in the rescuing cluster were more likely to blame other people or circumstances for their problems (lower SD1) and less certain about their goals in life (SD2) than the others in the same cluster (see Fig. 3C). Therefore, the lower life satisfaction of these individuals is associated with their being less well engaged and less certain about their chosen specialty or about their goals and circumstances in life. In our cross-sectional study we cannot determine whether their personality reduced their life satisfaction or their life satisfaction altered the aspects of their personality. Nevertheless, neither of these possibilities is compatible with the importance of personality-specialty congruence that we had originally hypothesized, but is consistent with a hypothesis of self-directedness promoting life satisfaction through success in goal-attainment (Judge et al., 2005). The general predisposition to plasticity and resilience in physicians (which is derived particularly from high Self-directedness along with high Persistence and low Harm Avoidance) appears to be more important for subjective well-being than person-environmental matching.

We hope that with longitudinal studies of larger samples will allow us to evaluate the relative roles of antecedent personality and job stressors on physician well-being. Increasing physician’s self-awareness of their own personality can promote character development and in turn their health, resilience, and well-being (Campanella et al., 2014; Cloninger & Cloninger, 2011; Krasner et al., 2009).

### Limitations

We had several issues that limit the scope of our findings. The response rate was low which was expected since there was no benefit to an individual for participating. There is likely a sampling bias with participation from only the most cooperative people or from people with a more positive outlook on psychiatric research. Participation varied greatly by specialty. We do not have data on non-responders or know what prevented them from participating. We did not identify any clusters with high HA which likely means there is a more cautious cohort that did not participate.

We identified five clusters but we do not know the proportionality of them in each field. We could be missing additional clusters of physicians hidden in those physicians who did not respond. Gender differences in personality likely play a role in specialty choice and could influence cultural differences seen between specialties (Reed, Jernstedt & Reber, 2001; Vaidya et al., 2004).

We only surveyed trainees at one hospital, which has its own unique culture for each specialty and may not generalize to other training sites. Future studies with larger sample sizes could get more information on subspecialties and the specialties with smaller overall populations. Longitudinal studies will be essential to distinguish cause from effect underlying the associations between personality and life satisfaction.

### Conclusions

We confirmed prior work indicating that physicians as a group are high-functioning individuals who are usually adaptable and resilient as indicated by their being high in self-directedness, cooperativeness, persistence, and life satisfaction. Here we found that there is substantial heterogeneity among physicians beyond these core traits and that this heterogeneity in personality profiles is strongly associated with individual differences in specialty choice among medical residents at the same teaching hospital. Our findings demonstrate the strong but complex relationships between personality profile, medical specialty choice, and well-being. A person’s personality profile appears to be strongly related to their choice of medical specialty in ways that are consistent with prior observations. We also found that physicians who chose pathology or emergency medicine as their specialty had lower life satisfaction if their own profile was congruent with that typically associated with that specialty.

There are two practical implications of these findings for medical education and training at the present time. Given the recent emphasis on selecting medical students for psychosocial strengths as well as intellect, we demonstrate that there are some core traits related to plasticity and resilience that can be identified, particularly being highly self-directed, cooperative, and persistent. Given the concern about high rates of burn-out and stress in medical students, residents, and practicing physicians, it will be important to evaluate the effect of matches in personality profile and specialty demands with the well-being of physicians in training. This will require testing entire cohorts of trainees and following them prospectively. What we can say now is that people with diverse personality profiles can adapt well to the different demands of various medical specialties. Given this diversity, it is likely to be more productive to focus on health promotion of medical students and physicians than on selectively matching people to particular specialties. In other words, all physicians can benefit from programs that promote their recognition of how their own personality and skills may be adapted to the demands of different specialties. Such programs can promote the health and well-being of physicians by cultivating their self-awareness and skills in emotion self-regulation and respectful emotional communication.

##  Supplemental Information

10.7717/peerj.2319/supp-1 Table S1 Distribution of TCI subscales across subjects and specialties (all 169 subjects)

10.7717/peerj.2319/supp-2 Table S2 Associations between clusters of subjects sharing TCI subscales and specialties

## Additional Information and Declarations

### Data Availability

The following information was supplied regarding data availability: The raw data has been supplied as Supplemental Datasets.
